# Extension of the standard addition method by blank addition

**DOI:** 10.1016/j.mex.2015.09.001

**Published:** 2015-09-09

**Authors:** Panagiotis Steliopoulos

**Affiliations:** CVUA Karlsruhe, Weißenburgerstraße 3, 76187 Karlsruhe, Germany

**Keywords:** Standard addition, Blank addition, Quantification, Chemical analysis

## Abstract

Standard addition involves adding varying amounts of the analyte to sample portions of fixed mass or fixed volume and submitting those portions to the sample preparation procedure. After measuring the final extract solutions, the observed signals are linearly regressed on the spiked amounts. The original unknown amount is estimated by the opposite of the abscissa intercept of the fitted straight line [1]. A limitation of this method is that only data points with abscissa values equal to and greater than zero are available so that there is no information on whether linearity holds below the spiking level zero. An approach to overcome this limitation is introduced.

• Standard addition is combined with blank addition.
• Blank addition means that defined mixtures of blank matrix and sample material are subjected to sample preparation to give final extract solutions.
• Equations are presented to estimate the original unknown amount and to calculate the 1-2α confidence interval about this estimate using the combined data set.

## Graphical abstract

**Figure:**
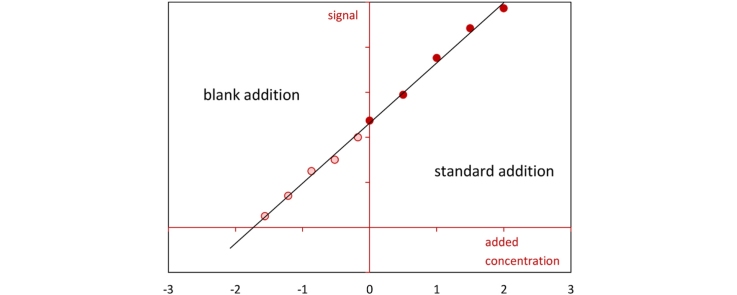

## Method details

Suppose that a liquid sample contains a particular compound of interest at unknown concentration. To determine this concentration, *n* aliquots of the sample are spiked with the analyte at concentration levels ${\left(x_{i}\right)}_{i=1,\ldots ,n}$. Furthermore, sample material is diluted with blank matrix to give mixtures with sample volume fractions of ${\left(k_{j}\right)}_{j=1,\ldots ,m}$ (ratios of sample volume to volume of total mixture). From each solution of the two series an aliquot of the same volume is taken and submitted to chemical analysis. The observed measurement values *y*_1_, …, *y*_*n*+*m*_ are considered as realizations of random variables *Y*_1_, …, *Y*_*n*+*m*_. Our statistical model is

(1) Y=Xβ+ε

where

$$\text{Y}=\left(\begin{array}{c} Y_{1}\\ \vdots \\ Y_{n}\\ Y_{n+1}\\ \vdots \\ Y_{n+m} \end{array}\right),\;\text{X}=\left(\begin{array}{cc} 1 & x_{1}\\ \vdots & \vdots \\ 1 & x_{n}\\ k_{1} & 0\\ \vdots & \vdots \\ k_{m} & 0 \end{array}\right),\;\beta =\left(\begin{array}{c} \beta_{0}\\ \beta_{1} \end{array}\right)=\left(\begin{array}{c} \beta_{1}x^{*}\\ \beta_{1} \end{array}\right)$$

and

$$\varepsilon =\left(\begin{array}{c} \varepsilon_{1}\\ \vdots \\ \varepsilon_{n}\\ \varepsilon_{n+1}\\ \vdots \\ \varepsilon_{n+m} \end{array}\right)$$

x^*^ designates the unknown initial concentration of the analyte. The parameter *β*_1_ is the slope and the parameter *β*_0_ is the ordinate intercept of the straight line that relates the expectation of the signal to the spiked concentration. Note that *β*_0_ is equal to *β*_1_x*. Put another way, the functional relationship between the expectation of the signal and the totally present concentration is supposed to be given by a straight line through the origin. **ɛ** is the vector of the measurement errors. We assume that *ɛ*_1_, …, *ɛ*_*n*+*m*_ is a sequence of independent and identically normally distributed random variables with expectation value zero and variance *σ*^2^. Minimizing the sum of the squared deviations provides an estimator **b** for the parameter vector **β**:

(2) $$\text{b}=\left(\begin{array}{c} b_{0}\\ b_{1} \end{array}\right)={\left({\text{X}}^{\prime }\text{X}\right)}^{\prime \prime }{\text{X}}^{\text{′}}\text{Y}$$

that is

(3) $$b_{0}=\frac{\sum_{i}x_{i}^{2}\left(\sum_{i}Y_{i}+\sum_{j}Y_{n+j}k_{j}\right)-\sum_{i}x_{i}Y_{i}\sum_{i}x_{i}}{\sum_{i}x_{i}^{2}\left(n+\sum_{j}k_{j}^{2}\right)-{\left(\sum_{i}x_{i}\right)}^{2}}$$

and

(4) $$b_{1}=\frac{\sum_{i}x_{i}Y_{i}\left(n+\sum_{j}k_{j}^{2}\right)-\sum_{i}x_{i}\left(\sum_{i}Y_{i}+\sum_{j}Y_{n+j}k_{j}\right)}{\sum_{i}x_{i}^{2}\left(n+\sum_{j}k_{j}^{2}\right)-{\left(\sum_{i}x_{i}\right)}^{2}}$$

with *i* = 1, …, *n* and *j* = 1, …, *m* (*b*_1_ is an estimator for the slope *β*_1_ and *b*_0_ is an estimator for the ordinate intercept *β*_0_). An estimator for the originally present unknown concentration is

(5) $${\hat{x}}^{*}=\frac{b_{0}}{b_{1}}=\frac{\sum_{i}x_{i}^{2}\left(\sum_{i}Y_{i}+\sum_{j}Y_{n+j}k_{j}\right)-\sum_{i}x_{i}Y_{i}\sum_{i}x_{i}}{\sum_{i}x_{i}Y_{i}\left(n+\sum_{j}k_{j}^{2}\right)-\sum_{i}x_{i}\left({\sum_{i}Y_{i}}_{i}+\sum_{j}Y_{n+j}k_{j}\right)}$$

Note that by replacing the random variables *Y*_1_, …, *Y*_*n*+*m*_ with the corresponding observed values *y*_1_, …, *y*_*n*+*m*_, the above estimators become estimates. The abscissa values of the blank addition data points can be computed as

(6) $${\hat{x}}_{n+j}=(k_{j}-1){\hat{x}}^{*}$$

Un unbiased estimator for the variance *σ*^2^ is

(7) $$\begin{array}{ll} S^{2} & =\frac{{\left(\text{Y}-\text{Xb}\right)}^{\prime }(\text{Y}-\text{Xb})}{n+m-2}\\ \; & =\frac{1}{n+m-2}\left(\sum_{i}{\left(Y_{i}-b_{0}-b_{1}x_{i}\right)}^{2}+\sum_{j}{\left(Y_{n+j}-b_{0}k_{j}\right)}^{2}\right) \end{array}$$

Un unbiased estimator for the variance of the estimator *b*_0_ + *b*_1_*x*_0_ at a certain abscissa value *x*_0_ is

(8) $$\begin{array}{ll} S_{b_{0}+b_{1}x_{0}}^{2} & =S^{2}(1\;x_{0}){\left({\text{X}}^{\text{′}}\text{X}\right)}^{\prime \prime }\left(\begin{array}{c} 1\\ x_{0} \end{array}\right)\\ \; & =S^{2}\left(\frac{\sum_{i}x_{i}^{2}-2x_{0}\sum_{i}x_{i}+x_{0}^{2}\left(n+\sum_{j}k_{j}^{2}\right)}{\sum_{i}x_{i}^{2}\left(n+\sum_{j}k_{j}^{2}\right)-{\left(\sum_{i}x_{i}\right)}^{2}}\right) \end{array}$$

The limits of a 1 − 2*α* confidence interval for *β*_0_ + *β*_1_*x*_0_ are

(9) $$L_{\text{upper/lower}}=b_{0}+b_{1}x_{0}\pm t_{1-\alpha ,n+m-2}S_{b_{0}+b_{1}x_{0}}$$

where *t*_1−*α*,*n*+*m*−2_ denotes the 1 − *α* quantile of the *t*-distribution with *n* + *m* − 2 degrees of freedom. Setting *L*_upper/lower_ equal to zero and solving for *x*_0_ yields the limits of a 1 − 2*α* confidence interval for the abscissa intercept $-\frac{\beta_{0}}{\beta_{1}}$. The corresponding opposites are the limits of a 1 − 2*α* confidence interval for the unknown initial concentration $x^{*}=\frac{\beta_{0}}{\beta_{1}}$:

(10) $$L_{x^{*},\text{upper/lower}}=\frac{b_{0}b_{1}+{\left(tS\right)}^{2}q\pm tS\sqrt{b_{0}^{2}r+b_{1}^{2}p+2b_{0}b_{1}q+{\left(tS\right)}^{2}(q^{2}-rp)}}{b_{1}^{2}-{\left(tS\right)}^{2}r}$$

with

$$q=\frac{\sum_{i}x_{i}}{\sum_{i}x_{i}^{2}\left(n+\sum_{j}k_{j}^{2}\right)-{\left(\sum_{i}x_{i}\right)}^{2}}\text{,}$$

$$r=\frac{n+\sum_{j}k_{j}^{2}}{\sum_{i}x_{i}^{2}\left(n+\sum_{j}k_{j}^{2}\right)-{\left(\sum_{i}x_{i}\right)}^{2}}\;\text{and}\;p=\frac{\sum_{i}x_{i}^{2}}{\sum_{i}x_{i}^{2}\left(n+\sum_{j}k_{j}^{2}\right)-{\left(\sum_{i}x_{i}\right)}^{2}}$$

**Fig. 1:**
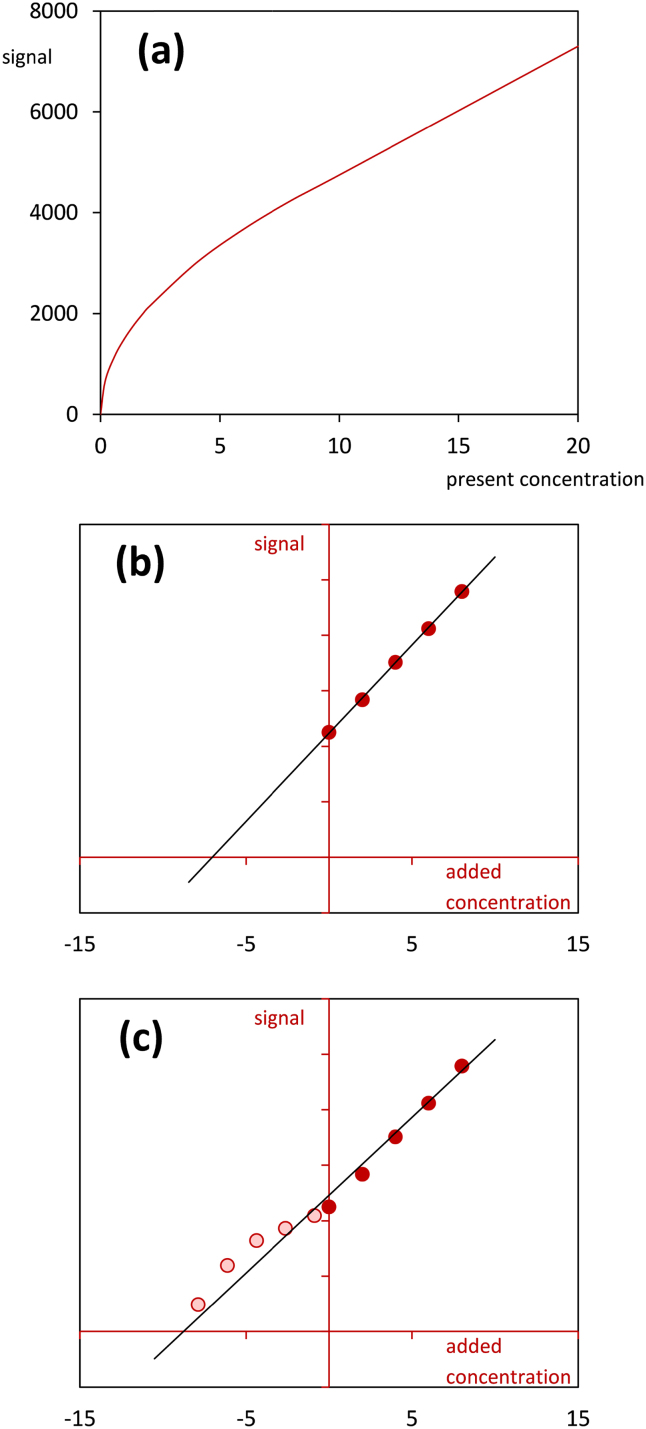
An illustration of what might happen if nonlinearity occurs below the spiking level zero.(a)Assumed nonlinear relationship between the expected signal and the present concentration.(b)Standard addition.(c)Standard addition combined with blank addition.

**Fig. 2:**
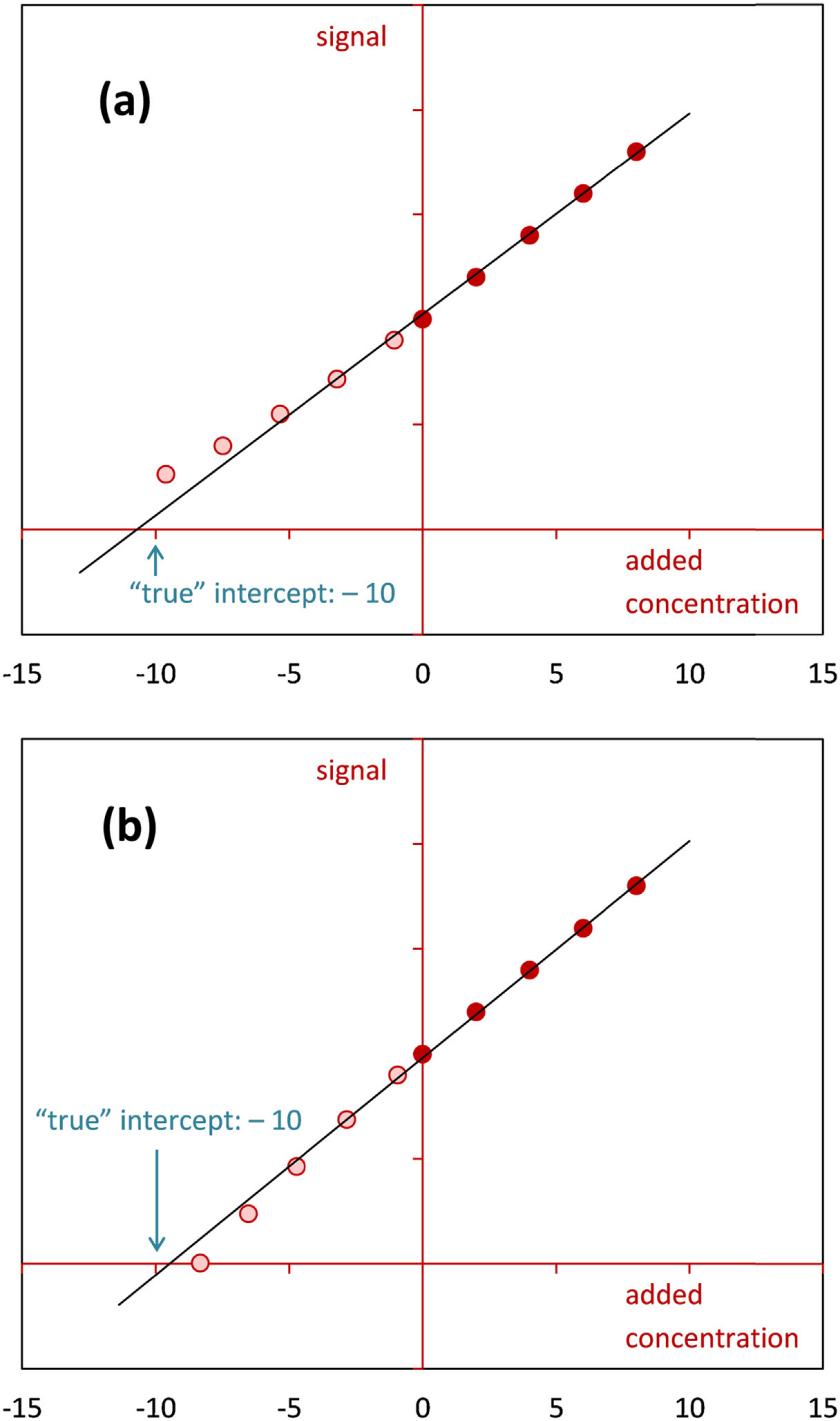
An illustration of what might happen if standard addition is combined with blank addition, although the sample matrix and the added blank matrix exhibit different matrix effects.(a)The slope that relates the expected signal to the present concentration is greater in the case of the sample matrix than in the case of the matrix used for blank addition.(b)The slope that relates the expected signal to the present concentration is smaller in the case of the sample matrix than in the case of the matrix used for blank addition.

**Fig. 3:**
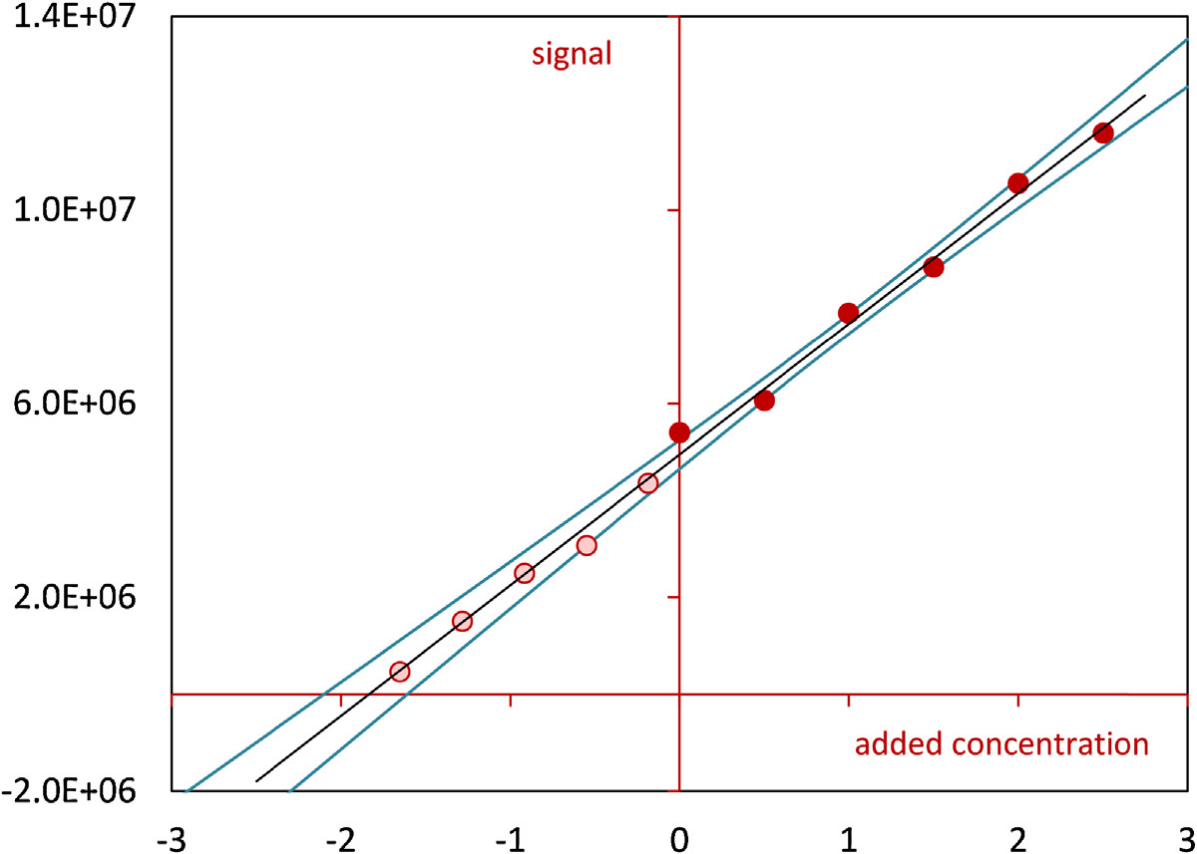
Quantification of nandrolone in a bovine urine sample by standard addition combined with blank addition (plot of the standard and blank addition line, the 0.95 confidence bands and the data points).

The advantage of the extended approach described in this paper over the classical standard addition method [1] is that the former allows to see whether linearity holds below the spiking level zero. For illustration, consider the following example. Suppose that the expected signal is related to the present concentration by the curve shown in Fig. 1(a). Analysis of a sample which contains the analyte at a concentration of 2 μg/L using the standard addition method gives the line depicted in Fig. 1(b) and a result of 7.0 μg/L. Although the standard addition plot looks quite good, the result considerably exceeds the actual concentration. Combining standard addition with blank addition (Fig. 1(c)) leads to a result of 8.8 μg/L, which is even higher, but now it is obvious that the outcome is erroneous because the blank addition data points clearly deviate from the fitted straight line and follow a curve. A basic assumption of the combined approach is that the sample matrix and the added blank matrix cause the same matrix effect; or, mathematically speaking, the relationship between the expected signal and the present concentration for both matrices is given by the same line through the origin. Fig. 2 shows two situations in which the model Eq. (1) is fitted to a standard and blank addition data set that does not fulfill this requirement. As it can be seen from this figure, when the slope that relates the expected signal to the present concentration is greater in the case of the sample matrix than in the case of the matrix used for blank addition, then the combined approach will tend to overestimate the unknown initial concentration. Conversely, when the slope that relates the expected signal to the present concentration is smaller in the case of the sample matrix than in the case of the matrix used for blank addition, then the combined approach will tend to underestimate the unknown initial concentration. The application of the combined approach is not only restricted to liquid matrices. Some examples from the laboratory of the author where the combined approach proved to be suitable are: quantification of steroids in urine by LC–MS, quantification of antibiotic residues in animal tissues (muscle and kidney) by LC–MS and quantification of artificial sweeterners in mineral water by LC–MS. Table 1 and Fig. 3 summarize the results of the analysis of a male bovine urine sample for its content of the steroid nandrolone by an LC–MS assay. A series of six aliquots of the urine sample were spiked with the target analyte at levels of 0, 0.5, 1.0, 1.5, 2.0 and 2.5 μg/L. Additionally, dilutions of the urine sample with nandrolone-free urine were prepared at sample volume fractions of 0.1, 0.3, 0.5, 0.7 and 0.9. Table 1 lists the observed signals, the estimated parameters, the estimate for the originally present unknown concentration, the 1 − 2*α* confidence interval for the originally present unknown concentration and the calculated abscissa values of the blank addition data points. For comparison, both the combined approach and the standard addition method were applied to quantify nandrolone. The two methods provided similar estimates. The smaller confidence interval obtained with the extended method is due to the higher number of data points used. Fig. 3 displays the data points, the straight line fitted to the combined data set and the 0.95 confidence bands.

## Figures and Tables

**Table 1:** Quantification of nandrolone in a bovine urine sample.

Data				Quantification by standard addition combined with blank addition					Quantification by standard addition			
*x*_*i*_	*y*_*i*_	*k*_*i*_	*y*_*n+j*_	*b*_0_	*b*_1_	${\hat{x}}_{n+j}$	${\hat{x}}^{*}$	0.95 confidence interval	*b*_0_	*b*_1_	${\hat{x}}^{*}$	0.95 confidence interval
0.0	5394708	0.1	464139	4950060	2696536	−1.65	1.84	[1.61; 2.10]	4140247	2592797	1.98	[1.56; 2.54]
0.5	6061705	0.3	1497300			−1.29						
1.0	7872740	0.5	2492913			−0.92						
1.5	8822356	0.7	3069726			−0.55						
2.0	10548385	0.9	4348496			−0.18						
2.5	11587569											

*n* = 6, *m* = 5, combined approach: *t*_1−0.25_ = 2.26 (9 degrees of freedom), standard addition: *t*_1−0.25_ = 2.78 (4 degrees of freedom).
